# Commencements

**Published:** 1897-08

**Authors:** 


### COMMENCEMENTS.

### Chicago College of Dental Surgery

The fifteenth annual commencement of the Dental Department of Lake Forest University, was held in Central Music Hall, Chicago, Thursday, April 1st, 1897, at 2 P. M.

The exercises opened with an invocation by Rev. Wm. M. Lawrence. An address was delivered by John J. Halsey, M.A., acting President of the University. The annual report was given by A. W. Harland, A.M., M.D., D.D.S., Secretary. The class valedictory was given by E. A. Rhyer. The doctorate address was delivered by L. L. Skelton, A.M., M.D.

The degree of D.D.S., was conferred by Dr. Truman W. Brophy, M.D., D.D.S., LL.D., President of the College, upon the following graduates:

Oliver Spencer Applegate
Alfred Ed D Austin
James Albert Atchison
Elmer Curtis Burkholder
Frank Clinton Babcock
Edwin Angelo Billig
David Newell Boatner
Edgar Robert Bennecke
William Porter Brown
Carl DeWitt Bates
Charles Henry Broadway
Ernst Oscar Bloomer
Bertis Bee Bemis
Washington Buchanan
Evan Henry Bryan
James Bryan Baker
Henry Brown
Guy Thomas Brearley
William Bebb
Charles Hiram Blackburn
Benjamin F Blosser
Leon Gilford Borst, M.D.
Claude Allison Costley
Perry Lee Cambell
George Albert Cutteridge
Charles H Cutteridge
Franklin Oliver Cates
Lorwin Noah Cates
Frederick H Colter
Edwin Monroe Clotfelter
Frank Brunner Clemmer
Howard S Clemmer
Martin Christensen
Clifford Warren Carr
Gustav Everett Cleophas
John Everett Clark
John Ward Corbin, Ph. C.
Otis Edward Chappell
Charles W Cotton
Charles Arthur Christin
Harry Clifford Devereaux
Harry Louis Dickinson
John Sweet Donald, B. Sc.
William Edmund Dunbar
Ernest George Downey
Frederick De Bruin
James Oregon Dunn
Christian F E Dreibrodt
Blake William Dutton
Milton Eisenstaedt
Charles Durbin Eversole
Anthony L Entsminger
Solomon Berman Friend
Henry Ember Fowler
Harry James Glasgow
Haakon Leonard Gerner
Warren A Giddings
Guy Boyce Harvey
Fred L Hasbrouck
Stephen R Harrison, Ph.B.
Charles N Hoagland
Grier Hanson
Irving James Herrick
Robert M Hettinger
Harold Hawthorne Hayes
Thomas Henry Hood
Morris Lincoln Hilton
Robert Henry Huffman
Louis S Irgens, Ph. G.
Charles Beach Ingram
John Harvey Johnston
Charles Abraham Kaye
August Frederick Kemper
Willett Edgar Kyle
Bert Amos Kimball
Robert Francis Livingston
Alfred Walter Lane
Henry C Loppenthien
William G Loppenthien
Walter B Lockwood, B. S.
Mahlon Ramsie Lindley
George Alfred Miller
Charles Gardner Morrell
George Edward Mason
Louis Hiram Matter
Valentine Massman
Martin D Molitor
John Laurence Malone
Francis N Maginnis
Frederick James Martin
Harry Irving Miner
Lantson Daniel Mills
Thomas Scott McAyeal
Erwin James McKee
George O Orr, D. V. S.
Edward F O'Donnell
Fred B Olwin
Maurithz Walter Olson
James Harvey Pearson
William Leaton Pank
Harry Hume Porter
Arthur Israel Porges
Harrie Allen Penfield
Joseph Carl Pasqueth
Joseph Loran Pease
Price Walton Rood
Charles Abraham Raver
Frederick H Roberts
Stanley Rea
Charles Frederick Rodolf
Edward B Rhinehart
Enoch Arden Reyher
Henry Warner Rich
George Edgar Stevenson
Harry Thurman Silver
Henry Stephen Smith
Emil T Strongquist
John Franklin Sweeney
John Martin Singler
Oscar Irving Spitz
Brace David Schrantz
Thomas Euell Stoddert
Henry Dee Single
Burton Schrock
Josiah Bridges Solomon
W M Standish, D. V. M. S.
Joseph Satory
Hezekiah M Thatcher
Raymond O Trenholm
Delos Bennett Terry
Fred Alonzo Tate
William McConnell Terry
Ross Sylvester Vedder
Edgar Randolph Weart
Lewis Edgar Wood
Martin Williams
John Liephart Wetzel
Robert Henry Wagner
George W Wilson
Richard T Woollard
Julian Emerson Young
John Matthias Yahres

### Philadelphia Dental College.

The thirty-fourth annual commencement exercises of the Philadelphia Dental College were held at the Academy of Music, Philadelphia, Pa., on Friday, April 2, 1897, at 12 o'clock M.

The address to the graduates was delivered by Professor S. H. Guilford, A.M., D.D.S., Ph.D., and the valedictory address by Lincoln A. Timerman, D.D.S.

The number of matriculates was two hundred and sixty-nine.

The degree of D.D.S. was conferred on the following graduates by Ex-Governor James A. Beaver, president of the board of trustees:

| NAME. | RESIDENCE. |
| --- | --- |
| Gustav N Aasgard | Norway |
| George F Ames | California |
| George D Arnum | Canada |
| George S Bailey | Pennsylvania |
| Arthur G Beach | Connecticut |
| George L Bean | California |
| Louis F Bean | Massachusetts |
| E M Bernardy | Pennsylvania |
| Edward B Bowker | Australia |
| Wilber H Brackett | Maine |
| Emma L Brecheisen | Ohio |
| John D Brennan | Pennsylvania |
| Albert C Bridgland | Canada |
| F T Bridgland | Canada |
| William E Brown | New York |
| William J Brown | Canada |
| William H Bulmer | Canada |
| Mitchell H Burke | Massachusetts |
| Dan U Cameron | Ohio |
| Wm M Cameron | Canada |
| Jerome C Capron | New York |
| William S Carnes | Ohio |
| William A S Casey | Canada |
| Isaac P Cliff | Australia |
| Centennella Collins | Pennsylvania |
| John G Colridge | Canada |
| William R Conner | Canada |
| Edward J Counter | Australia |
| Walter W Crate | New Jersey |
| S A Crowther | Pennsylvania |
| George J De Long | Pennsylvania |
| W E Dickson | Massachusetts |
| Daniel J Dixon | Massachusetts |
| Frank J Doland | Massachusetts |
| W H Dolman | California |
| Joseph R Dunbar | Pennsylvania |
| Harry C Easton | Pennsylvania |
| Henry A Eltz | Pennsylvania |
| Frank J Erbe | Connecticut |
| R McE Erwin, Jr | Pennsylvania |
| Harry E Fitch | New York |
| Stanley Fitz Stubbs | Australia |
| John T Frederick | Pennsylvania |
| J C Gilbertson | California |
| George M Glenney | Canada |
| Edwin A Goodwin | Canada |
| Herman J Graham | New York |
| Edward P Grove | Pennsylvania |
| Louis E Habegger | Pennsylvania |
| John W Hall | England |
| Louis W Hart | Maine |
| Wm H Harvey | California |
| Charles P Heales | Canada |
| Samuel E Heilig | Pennsylvania |
| W T S Hinder | Australia |
| James H Holtham | Pennsylvania |
| Willard Hoster | New York |
| F H Hauck | Oregon |
| Cromwell Ironside | New Jersey |
| James W Jackson | New York |
| Willis H Jackson | New York |
| John A Johnson | Pennsvlvania |
| Walter M Johnson | Canada |
| Chas H Kaufman | Illinois |
| Ralph H Keeler | Connecticut |
| George W Kleiser | California |
| E E Kinney, M S | Canada |
| C A Kirkpatrick | Canada |
| Edward N Lambly | Canada |
| Frank C Lewis | Pennsylvania |
| Chas T Lipscomb | South Carolina |
| F McL Longley | Georgia |
| Edward N Lord | New York |
| Burt A Loveless | New York |
| Frank W Lowrey | Maine |
| Jas W McClune | Pennsylvania |
| E M McConaughy | Iowa |
| J E McIlwraith | Rhode Island |
| F H McLaughlin | Canada |
| Peter E Mackay | Canada |
| Armand G Mader | Pennsylvania |
| M W Maloney | Massachusetts |
| John J Mathias | Pennsylvania |
| John J Mattison | New York |
| Fred E Maxfield | Maine |
| James J Mills | Pennsylvania |
| Stuart McL Milne | Canada |
| Hal H Mize | Missouri |
| Lloyd G Morgan | Oregon |
| M W Morris | Canada |
| Mary A Morrison | New Jersey |
| Herbert S Nelson | Pennsylvania |
| Wm S O'Brien | New York |
| Wm E O'Reilly | Massachusetts |
| Adin T Parsons | Australia |
| E A Pennington | New Jersey |
| Albert E Phin | Canada |
| S L Pickles | Canada |
| Chas H Pinkham | New Jersey |
| Catharine S Platt | New Jersey |
| M Propper, M D | Hungary |
| Woodie S Pugh | Alabama |
| Maurice W Quinn | Massachusetts |
| Richard H Rivers | Canada |
| Albert L Roat | Pennsylvania |
| Wm F Roberts | Rhode Island |
| Moses N Roe | New York |
| Chas S Roever | Maryland |
| E S Rosenbluth | Connecticut |
| E Schnoll, M D | Austria |
| A Schreier, M D | Austria |
| Herbert W Shaw | Massachusetts |
| James M Shay | New York |
| Wm P Smedley | Colorado |
| David A Smith | New York |
| Robert E Smith | California |
| W F Sommers | Pennsylvania |
| Joseph W Steiner | Roumania |
| T C Stellwagen, Jr | Pennsylvania |
| Arthur J Sullivan | Indiana |
| Frank E Thomas | Iowa |
| Edna M Thompson | Ohio |
| Mattie S Tilton | New York |
| L A Timerman | New York |
| George S Wagner | Pennsylvania |
| J S Ware | New Jersey |
| George L Webb | Pennsylyania |
| Frank W Webster | Canada |
| E S White, B S | North Carolina |
| P B Whitmarsh | Rhode Island |
| Arthur D Williams | Canada |
| Walter J Williams | Canada |
| Reid A Wilson | Pennsylvania |
| Ralph A Wood | Maine |
| Ora E Zuill | Bermuda |

### Pennsylvania College of Dental Surgery.

The forty-first annual commencement exercises of the Pennsylvania College of Dental Surgery were held at the Academy of Music, Philadelphia, Pa., on Thursday evening, April 1, 1897.

The address to the graduates was delivered by Professor A. P. Brubaker, M. D.

The number of matriculates for the session was three hundred and sixty-two.

The degree of D.D.S. was conferred on the following graduates by I. Minis Hayes, M. D., president of the college:

| NAME. | RESIDENCE. |
|---|---|
| J A Anderson | New York |
| Jos H A Barretto | Pennsylvania |
| Earle T Beale | Pennsylvania |
| E J Beers | Pennsylvania |
| Adolphus Belber | Pennsylvania |
| H M Boyer | Pennsylvania |
| D N Booth | New Hampshire |
| Robt B Bryant | New York |
| Otto F Brauns | Wisconsin |
| Geo C Byrne | Pennsylvania |
| W M Campbell | New York |
| J G Cannon | Delaware |
| Frank Carroll | Maryland |
| R C Chambers | Pennsylvania |
| G O Challinor | Pennsylvania |
| J Roy Cherry | Pennsylvania |
| Annie F Colley | Maryland |
| S C Coleman | Canada |
| J D Coney | Pennsylvania |
| H A Coykendall | New Jersey |
| Jos F Crandall | Pennsylvania |
| R P Eas n | Georgia |
| Geo W Fleming | Pennsylvania |
| Edwin D Forrest | New Hampshire |
| Dionsio de Frias | San Domingo |
| C H Fraiser | New York |
| Lillian M Gale | Wisconsin |
| R G Garvin | Ireland |
| T E Gifford | New Jersey |
| W S Haliett | Delaware |
| R J Hawkey | Canada |
| C E Hart | Ohio |
| Wm Haverkost | Pennsylvania |
| A E Haynen | Canada |
| A M Hendershot | Ohio |
| W S Herr | Pennsylvania |
| E S Hine | New Jer ey |
| Francis T Houston | Ohio |
| Otto Homberger | Switzerland |
| J Max Hootman | Pennsylvania |
| J H Kalbach | Pennsylvania |
| Frank Kampe, Jr | Pennsylvania |
| Silas B Keith | Massachusetts |
| Arthur Koester | Germany |
| John A Lambertus | Canada |
| H H Lattimore | Georgia |
| W J Lawson | Ohio |
| F C Leffmann | Pennsylvania |
| R W Livingston | Pennsylvania |
| E S McDonald | Ohio |
| J W Manon | Pennsylvania |
| W J Martin, M D | Pennsylvania |
| R Mathison, Jr | Canada |
| G de Mazarredo | Cuba |
| F L Mills | Pennsylvania |
| R W Morgan | Pennsylvania |
| T W Morgan | Pennsylvania |
| J A Moran | Canada |
| J Fred Moore | Pennsylvania |
| L J Moulton | New Hampshire |
| Jas V Mulford | Ohio |
| Yasu Nakamura | Japan |
| Maude Nicholson | Pennsylvania |
| A J Norton | New York |
| H J Oldstein | Pennsylvania |
| L D Palmer | Pennsylvania |
| Geo F Paine | New York |
| Wilbur M Peirce | Pennsylvania |
| Robt Purvis | Washington, D C |
| G B Renfrow | Kentucky |
| J C Salvas | Wisconsin |
| H S Sanders | Pennsylvania |
| Wm C Scott | Pennsylvania |
| H L Schooley | Pennsylvania |
| R Ogden Shoop | Ohio |
| R G Skillen | California |
| R A Smith | Pennsylvania |
| H T Smith | Pennsylvania |
| Thos M Smith | New Jersey |
| H W Souders | Pennsylvania |
| M W Sommer | Pennsylvania |
| C Luco Solar | Chile |
| E O Spaulding | New Hampshire |
| L W Strong | Connecticut |
| A H Tait | Jamaica |
| S E Tate | Pennsylvania |
| C K Taylor | Pennsylvania |
| M J Terry | Pennsylvania |
| J C Todd | Pennsylvania |
| W E Towne | Massachusetts |
| Harry Van Allen | Canada |
| J Y Walker | Ohio |
| Oscar Wayne | Pennsylvania |
| V R Westervelt | New York |
| H Belle Whitcomb | Pennsylvania |
| A R White | New Jersey |
| Jas G Whiteside | Ohio |
| A L Wilson | Canada |
| W C Wilson | Pennsylvania |
| Tullus Wright | Pennsylvania |
| Fred Zimmerman | Switzerland |

### State University of Iowa.

The twentieth annual commencement of the Dental Department of this institution was held in the Opera House, March 16th, 1897, at 7:00 o'clock P. M.

The address was delivered by Rev. J. E. Cathill, of Des Moines.

The degree of Doctor of Dental Surgery was conferred by the Dean, Charles A. Schaeffer, Ph. D., upon the following candidates:

| NAME. | RESIDENCE. |
|---|---|
| Arthur Lewis Anderson | Estherville |
| George P Baughman | Griswold |
| Fred'k Channing Blanchard | Waterloo |
| Casper Milton Baltis Boos | Iowa City |
| Erwin Lester Burns | Kensett |
| George Edward Clark | Galesburg, Ills |
| Frank Conn | Cedar Rapids |
| Clarence Augustus Dodge | Burlington |
| Joseph Patrick Donlon | Elkader |
| Charles Sumner Fox | Meriden, Conn |
| Festus M Griffin | Missoula, Mont |
| Voclav O Hasek | Cedar Rapids |
| Frank Elmer Holland | Afton |
| Frederick Willis Horton | Iowa City |
| Norman H Hough | Muscatine |
| Frank Boynton James | Iowa City |
| John M Jones | Iowa City |
| Harry Carson Jones | Des Moines |
| Chas Rudolphus Kearns | Lincoln, Neb |
| Elmer Francis Kennedy | Cherokee |
| William B Liggett | Marcus |
| George R Leonard | Billings, Mont |
| Ernest George Lotts | Independence |
| Burlington J Maytum | Warsaw |
| Henry Morrow, Jr | Iowa City |
| Adam J Mueller | Iowa City |
| Carroll W Renshaw | Rock Valley |
| Gustavus Earnest Rizer | Fort Madison |
| Richard Erskine Scroggs | Indianola |
| Alson Secor | Forest City |
| John C Silvis | Rock Island |
| Roy F Smith | Mitchell, S D |
| F. Potter Smith | York, Neb |
| Samuel Breese Toney | Chicago, Ills |
| Charles Shuler Wilcox | Elgin, Ills |

### Tennessee Medical College, Knoxville, Dental Department.

The annual commencement of this department was held in April with the usual exercises.

The degree of Doctor of Dental Surgery was conferred upon the following candidates:

| NAME | RESIDENCE. |
|---|---|
| Garrett Allison Buckner | Tennessee |
| John Oramel La Cove | Michigan |
| Alexander Black Marbury | Missouri |
| Robert Andrew McCallie | Tennessee |
| Mathew Kyle Pennington | Kentucky |
| William Tandy Senter | Tennessee |
| James Frank Woodward | Virginia |

### Dental Department of Harvard University.

The commencement exercises of the Dental Department of Harvard University were held in conjunction with the other departments of the University in Sanders' Theatre, Cambridge, on Wednesday, June 30th, at 10:30 o'clock A. M., President Eliot conferring the degree of Doctor of Dental Medicine on the following candidates:

| | | |
|---|---|---|
| Fred Wild Allen | Walter Joseph Faunce | Joseph Thomas Mooney |
| Frank Pliny Barnard | George Lincoln Forrest | Charles E Parkhurst, A B |
| Roy Keney Belden | Leo Green, A B | Harry Carlton Spencer |
| Harry Ernest Belyea | George True Greenwood | J H Stromier, LDS, DDS |
| Charles Wm Berry, B S | Francis Herbert Harding | David Pickard Thomas |
| Ralph V Blake, Ph G | Edward Everett Henry | Clarence B Vaughan |
| Harold Edgeworth Davis | Arland Martin Kenney | Frank Hosea Veo |
| Robert Irving Davis | Charles Ansel Lakin | George Alfred Warren |
| Walter Sheldon Davis | William Lunan, Jr | Walter Harris White |
| John Dana Dickinson | Raimond E McDonnough | Herbert C Woodman |
| Harold Watson Estey | Thomas Rich'd McMahon | |

### Dental Department of University of Tennessee.

The annual commencement of this institution was held March 29th, 1897, at 8:00 o'clock P. M., in the Vendome Theatre.

The exercises were opened by a valedictory address to the class by T. B. King, of Tennessee. Cnarge to the class by Dr. John S. Marshall, of Chicago, after which the degree of Doctor of Dental Surgery was conferred upon the following candidates:

| NAME. | RESIDENCE. | NAME. | RESIDENCE. |
|---|---|---|---|
| James W Bryan | Tennessee | K J Schuman | Tennessee |
| W J Crutcher | Tennessee | M N Terrell | Alabama |
| T B King | Tennessee | S A Webre | Louisiana |
| W E Lundy | Mississippi | B W Vanamburg | Missouri |
| W O McIntire | Kentucky | W H Kemp | Tennessee |
| F C Williams | Virginia | L W Smith | Georgia |

